# DNGR-1 regulates proliferation and migration of bone marrow dendritic cell progenitors

**DOI:** 10.1084/jem.20241813

**Published:** 2025-05-13

**Authors:** Ana Cardoso, Michael D. Buck, Bruno Frederico, Probir Chakravarty, Oliver Schulz, Kok Haw Jonathan Lim, Cécile Piot, Mariana Pereira da Costa, Evangelos Giampazolias, Francesca Gasparrini, Neil Rogers, Caetano Reis e Sousa

**Affiliations:** 1 Immunobiology Laboratory, The Francis Crick Institute, London, UK; 2 Bioinformatics and Biostatistics, The Francis Crick Institute, London, UK

## Abstract

Conventional dendritic cells (cDCs) are sentinel cells that play a crucial role in both innate and adaptive immune responses. cDCs originate from a progenitor (pre-cDC) in the bone marrow (BM) that travels via the blood to seed peripheral tissues before locally differentiating into functional cDC1 and cDC2 cells, as part of a process known as cDCpoiesis. How cDCpoiesis is regulated and whether this affects the output of cDCs is poorly understood. In this study, we show that DNGR-1, an innate immune receptor expressed by cDC progenitors and type 1 cDCs, can regulate cDCpoiesis in mice. In a competitive chimera setting, cDC progenitors lacking DNGR-1 exhibit increased proliferation and tissue migratory potential. Compared with their WT counterparts, DNGR-1–deficient cDC progenitor cells display superior colonization of peripheral tissues but an altered distribution. These findings suggest that cDCpoiesis can be regulated in part by precursor cell-intrinsic processes driven by signals from innate immune receptors such as DNGR-1 that may respond to alterations in the BM milieu.

## Teaser

Cardoso et al. show that DNGR-1–deficient pre-cDCs display cell-intrinsic alterations in their ability to colonize peripheral organs. Their findings highlight an unexpected role for innate immune receptors in regulating cDCpoiesis.

## Introduction

Conventional dendritic cells (cDCs) are sentinel leukocytes that detect alterations in tissue environments and initiate immune responses against infection and cancer (Cabeza-Cabrerizo et al., 2021a; Guilliams et al., 2014; Merad et al., 2013). cDCs are found in all tissues but originate from a committed precursor in the bone marrow (BM), the common or conventional dendritic cell precursor (CDP). CDPs give rise to pre-cDCs that exit the BM via the bloodstream to seed tissues where they differentiate into “immature” type 1 and 2 cDCs (cDC1s and cDC2s) cells, a process known as cDCpoiesis (Bogunovic et al., 2009; Cabeza-Cabrerizo et al., 2021a; Guilliams et al., 2014; Liu et al., 2009; Merad et al., 2013; Varol et al., 2009). While the regulation of cDCpoiesis is incompletely understood, it is known that pre-cDCs recruitment to the periphery to support cDC turnover during homeostatic and stress (“emergency”) conditions is partially regulated by cytokine and chemokine signals (Cabeza-Cabrerizo et al., 2019, 2021b; Hochweller et al., 2009). For instance, cytokines, such as Flt3 (Fong et al., 2001; Maraskovsky et al., 1996; Naik et al., 2006; Salmon et al., 2016), and chemokines, such as C-chemokine ligand (CCL)2 (Cabeza-Cabrerizo et al., 2021b; Nakano et al., 2017; Pereira da Costa et al., 2023; Scott et al., 2015), impact pre-cDCs expansion in the BM and/or their exit into the blood and entry into tissues. Pre-cDCs can also directly respond to signs of infection or tissue damage as they express a wide array of innate immune receptors, in particular of the TLRs and C-type lectin receptor families (Cabeza-Cabrerizo et al., 2021b; Guermonprez et al., 2013; Nakano et al., 2017; Schmid et al., 2011; Singh et al., 2008). However, to what extent cell-intrinsic signaling by these receptors can control cDCpoiesis remains unclear.

Dendritic cell natural killer lectin group receptor-1 (DNGR-1), also known as C-type lectin domain family 9 member A (CLEC9A), is a C-type lectin receptor specifically expressed in the cDC lineage (Cabeza-Cabrerizo et al., 2021a; Cueto et al., 2020). In mice, it starts to be expressed by CDPs, which then differentiate into DNGR-1^+^ pre-DCs (Schraml et al., 2013). DNGR-1 expression is upregulated during pre-cDC to cDC1 differentiation but extinguished in cDC2s (Schraml et al., 2013). High expression on cDC1s but not cDC2s allows DNGR-1 to be used as a reliable marker for cDC1 in both mice and humans (Caminschi et al., 2008; Huysamen et al., 2008; Poulin et al., 2010, 2012; Sancho et al., 2008). Notably, besides acting as a cDC1 marker, DNGR-1 plays a fundamental role in cDC1 biology. DNGR-1 is an innate immune receptor that binds to filamentous actin (F-actin) exposed on dead cell corpses (Ahrens et al., 2012; Hanč et al., 2015; Zhang et al., 2012). In cDC1, signaling by DNGR-1 in response to ingested dead cell debris can lead to endosomal or phagosomal rupture (Canton et al., 2021). This allows access of dead cell–associated antigens to the cytosol of cDC1s to be processed and cross-presented to CD8^+^ T cells (Sancho et al., 2009). In contrast, the role of DNGR-1 in mouse cDC precursors, which, unlike cDC1, are not known for their cross-presenting ability, remains unclear. We therefore set out to investigate whether DNGR-1 expression by CDPs and pre-cDCs is functionally relevant for cDCpoiesis.

In this study, we demonstrate that DNGR-1 expressed by pre-cDCs can act in a canonical fashion to promote cross-presentation (XP) of dead cell–associated antigens. However, we also uncover a noncanonical role for the receptor in cDCpoiesis. We demonstrate that DNGR-1 expression in pre-cDCs impacts the expression of chemokines, receptors, and transcription factors involved in cell cycle regulation and in cell migration and adhesion. Consistent with the latter, the absence of DNGR-1 resulted in pre-cDCs that are more efficient than WT pre-cDCs at colonizing peripheral tissues but that disperse throughout those tissues rather than localize to specific niches. Our results suggest that DNGR-1 expression modulates cDCpoiesis and contributes to the correct localization of pre-cDCs in peripheral tissues.

## Results and discussion

### Low expression of DNGR-1 in cDC precursors can nevertheless facilitate XP of dead cell–associated antigens

**Figure 1.**
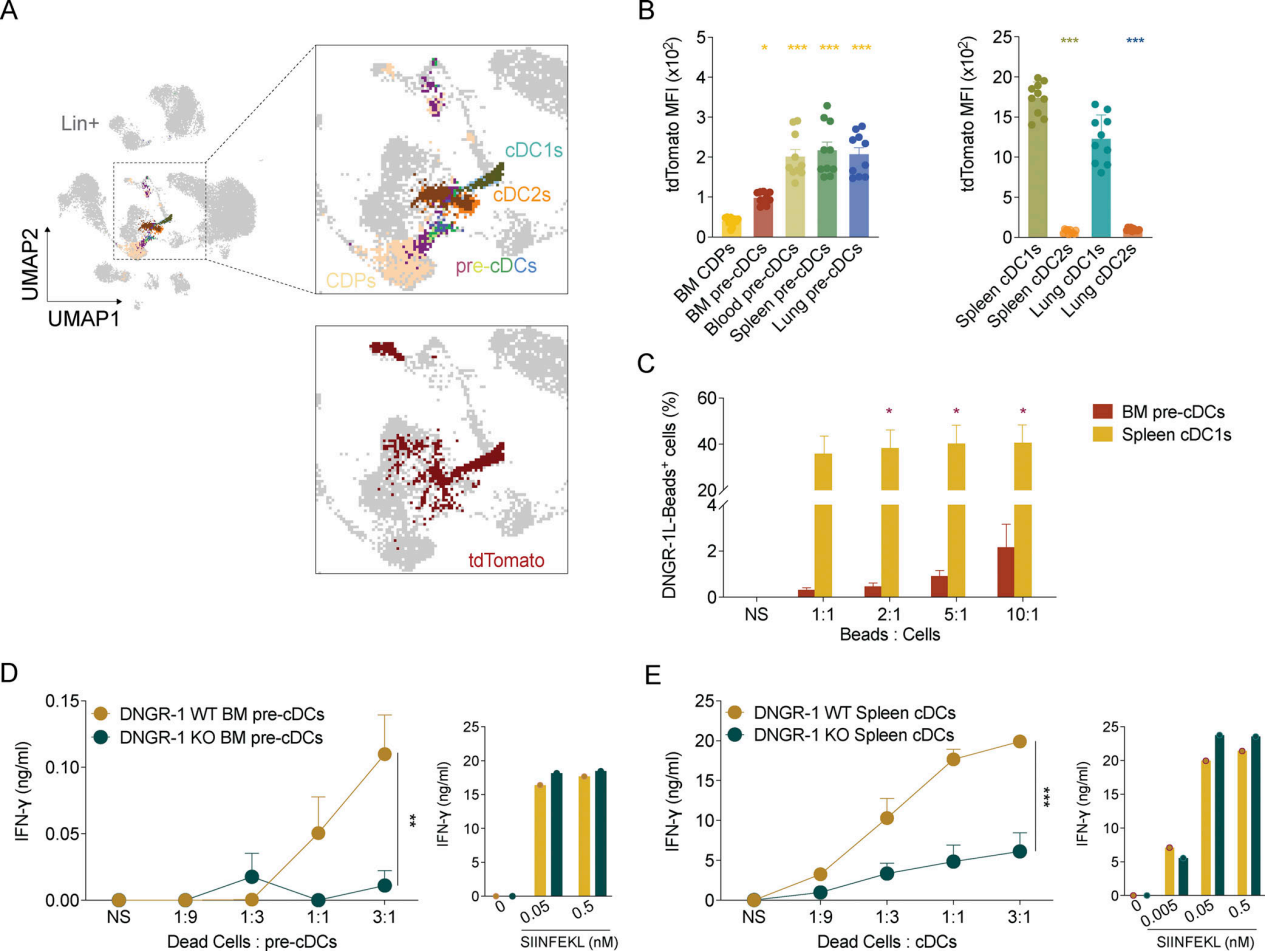
**Low levels of DNGR-1 expression by BM pre-cDCs suffice for mediating XP of dead cell–associated antigens. (A)** Uniform manifold approximation and projection (UMAP) analysis of flow cytometry data overlaying different immune populations, namely, effector CD45^+^ cells overlaid with different cDC lineage populations from BM, spleen, and lung, as identified before (Fig. S1, A–D) in *Clec9a*^*tdTom/tdTom*^ mice (data are from one mouse, representative of 10). **(B)** Mean fluorescence intensity (MFI) of tdTom expression in the indicated cell populations of *Clec9a*^*tdTom/tdTom*^ mice (*n* = 10). **(C)** WT BM pre-cDCs and splenic cDC1s were incubated with indicated ratios of DNGR-1 ligand (L)–coated beads to cells. Association of DNGR-1L–coated beads with BM pre-cDCs and splenic cDC1s was measured by flow cytometry. **(D and E)** (D) WT and DNGR-1 KO BM pre-cDCs or (E) splenic cDC1s were incubated with the indicated ratios of UVC-killed BRAF^V600E^ 5555 cells pulsed with OVA and poly(I:C) or with the indicated concentrations of SIINFEKL. NS represents non-stimulated. Pre-activated OT-I T cells were added and IFN-γ accumulation in culture supernatants was assessed by ELISA 16 h later. Each dot in B represents one mouse, and data are pooled from at least two experiments. Bars represent averages, and error bars represent SEM. Data in C–E depict averages from at least two experiments (total of three to five biological samples; each sample was pooled from two to three mice to achieve one biological replicate); error bars represent SEM. Statistical significance in B was calculated using one-way ANOVA, and in C–E two-way ANOVA with Sidak’s multiple comparisons test. *P < 0.05, **P < 0.01, and ***P < 0.001.

**Figure S1.**
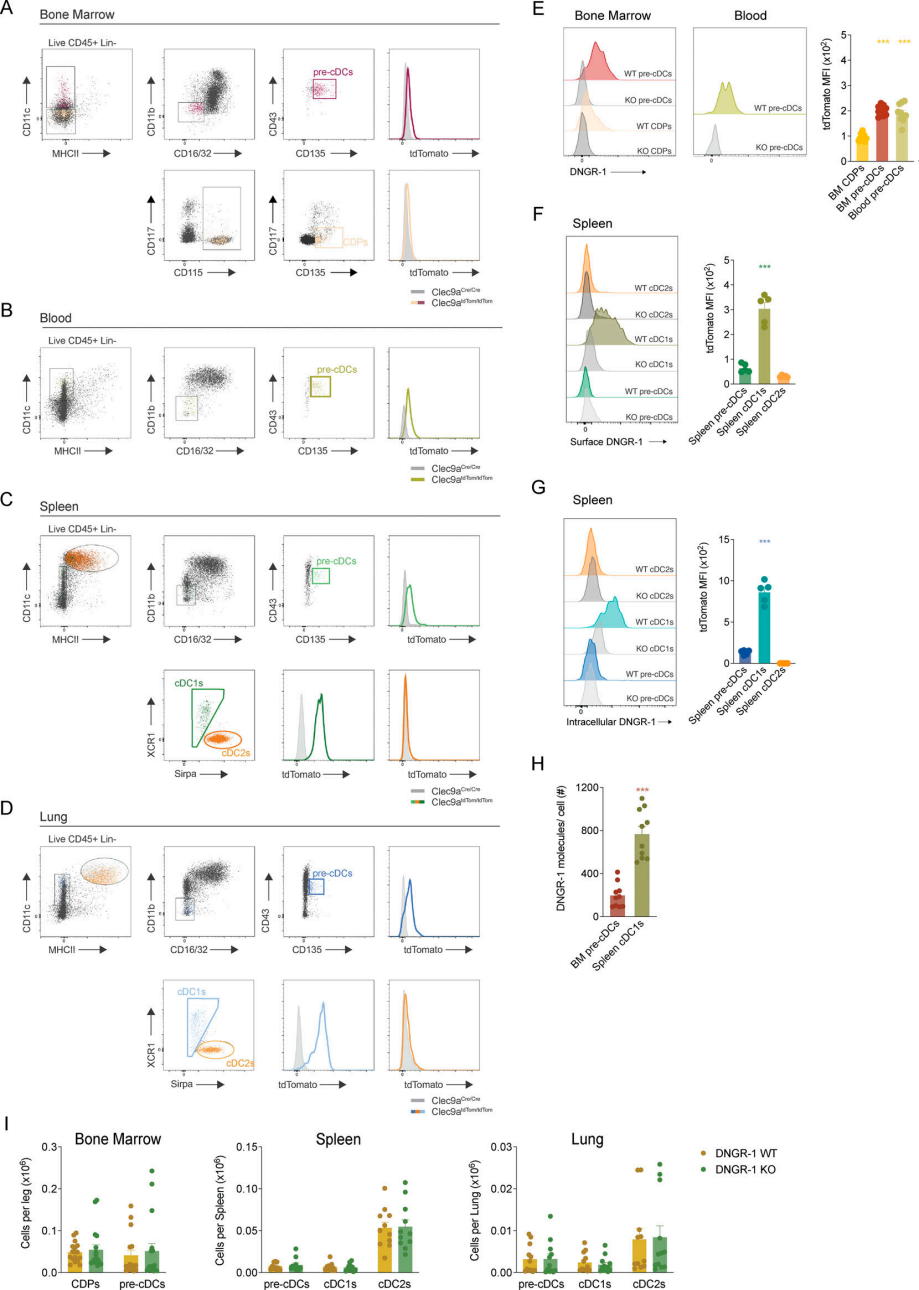
**FACS analysis of mouse cDCs and their progenitors. (A–D)** Gating strategy for CDPs, pre-cDCs, cDC1s, and cDC2s in (A) BM, (B) blood, (C) spleen, and (D) lung. All plots are pre-gated on live (live/dead dye negative) CD45^+^ lineage (Lin)-negative singlet cells. Lineage cocktail includes CD3, CD19, B220, NK1.1, Ly6G, Ly6D, Siglec-F, and Ter119. **(E)** Representative plots of anti–DNGR-1 staining (left) in CDPs and pre-cDCs from BM or blood. Right: intensity (MFI) of DNGR-1 staining on the indicated cell populations (*n* = 10 mice). **(F and G)** Representative plots of anti–DNGR-1 surface (F) or intracellular (G) labeling in spleen pre-cDCs and cDCs. Right: intensity (MFI) of DNGR-1 staining on the indicated cell populations (*n* = 5 mice). **(H)** Quantification of the number of DNGR-1 molecules per cell surface. **(I)** Quantification of total number of CDPs, pre-cDCs, and cDCs in the BM, spleen, and lung of adult WT (BL/6) and DNGR-1 KO (*Clec9a*^*Cre/Cre*^) mice (*n* = 11–17). Each dot in E, H, and I represents one mouse, and data are pooled from at least two experiments. Data in F and G are from one representative experiment out of three. Bars represent averages, and error bars represent SEM. Statistical significance was calculated in E–G using one-way ANOVA; H was calculated using an unpaired *t* test with Welch’s correction; and I was calculated using two-way ANOVA with Sidak’s multiple comparisons test. ***P < 0.001. MFI, mean fluorescence intensity.

To begin to address the function of DNGR-1 in CDPs and pre-cDCs, we first systematically measured levels of receptor expression in those cells after extraction from BM, spleen, or lungs. We made use of a *Clec9a*^*tdTom*^ mouse that reports *Clec9a* locus activity via tdTomato (tdTom) expression (Frederico et al., 2022) (Fig. 1, A and B; and Fig. S1, A–D). In parallel, we also stained cells with anti–DNGR-1 mAb, comparing intact and permeabilized cells to account for surface versus intracellular receptor pools (Fig. S1, E–G). Irrespective of the assay used, CDPs and pre-cDCs expressed substantially lower levels of DNGR-1 than differentiated cDC1s, consistent with a previous report (Schraml et al., 2013). Using quantitative flow cytometry analysis of stained intact cells (Fang et al., 2022), we estimate that BM pre-cDCs express ∼200 DNGR-1 receptors at the cell surface, while splenic cDC1 express around 800 (Fig. S1 H).

The low expression in cDC progenitors could limit the ability of DNGR-1 to function in detection of dead cells. Consistent with this possibility, only about 3% of BM pre-cDCs were able to bind and internalize DNGR-1 ligand-coated yellow green-fluorescent latex beads compared with up to 50% of WT splenic cDC1s (Fig. 1 C). However, surprisingly, pre-cDCs were still able to cross-present dead cell–associated antigens in a DNGR-1–dependent fashion. Indeed, BM pre-cDCs from C57BL/6 (WT mice) were superior to those from *Clec9*^*Cre/Cre*^ (DNGR-1 KO) mice at stimulating pre-activated OT-I CD8^+^ T cells when fed with necrotic cells soaked with ovalbumin and poly I:C (Fig. 1 D). However, XP activity by BM pre-cDC was far lower than that of splenic cDC1s used as positive controls in the same assay, as evidenced by an ∼130-fold difference in IFN-γ levels at the maximum dose of dead cell–associated antigen used (Fig. 1 E). This reduced activity is consistent with the lower expression of DNGR-1 in pre-cDCs compared with cDC1s (Fig. 1 B and Fig. S1, E–G) but may reflect additional differences, for example, lower uptake of dead cell debris. Of note, all cells were equally effective at stimulating OT-I when pulsed with subsaturating concentrations of the OVA-peptide SIINFEKL, demonstrating that DNGR-1 deficiency per se does not impact the overall ability of cDC progenitors or cDC1s to present antigen to CD8^+^ T cells (Fig. 1, D and E). Altogether, these findings indicate that DNGR-1 in BM pre-cDCs can execute canonical functions in dead cell detection and in promotion of XP of dead cell–associated antigens despite low levels of receptor expression. Whether XP of dead cell–associated antigens by pre-cDCs is functionally relevant in vivo remains to be assessed, as the levels of OT-I stimulation in the XP assay were exceedingly low. This may reflect a very low level of formation of SIINFEKL-H-K^b^ complexes per cell or, more likely, the activity of a very small fraction of pre-cDCs, perhaps corresponding to more differentiated cells that are committed to a cDC1 fate (Grajales-Reyes et al., 2015; Schlitzer et al., 2015). Further dissection of this issue was marred by the very small number of pre-cDCs that can be isolated for such XP assays. Nevertheless, our data indicate that DNGR-1, even at low expression levels, can execute canonical functions in pre-cDCs, as it does in cDC1s.

### DNGR-1 does not markedly affect pre-cDCs proliferation and differentiation

**Figure 2.**
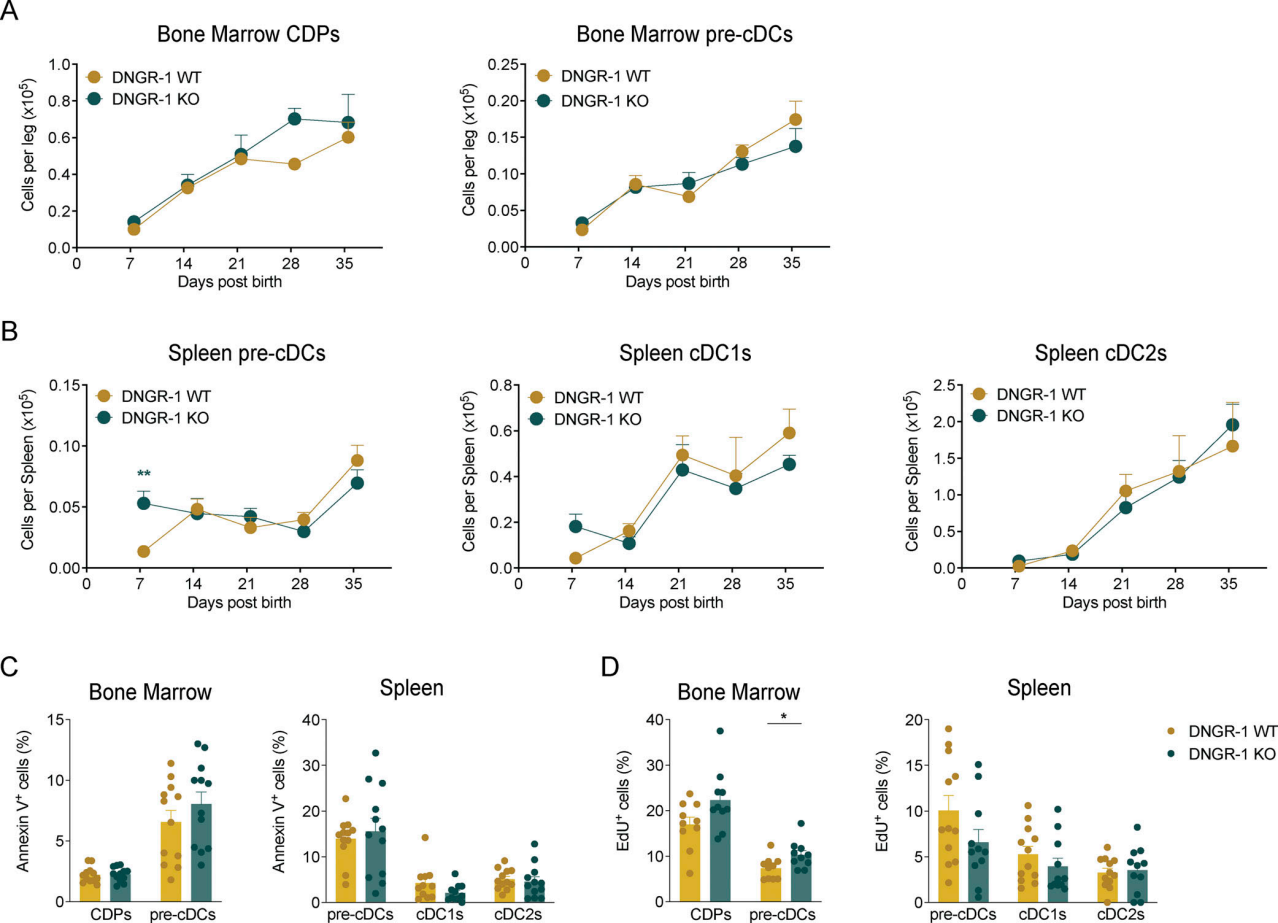
**DNGR-1 deficiency subtly affects the kinetics of the cDC network postnatal expansion. (A and B)** Quantification of total number of (A) BM CDPs and pre-cDCs and (B) spleen pre-cDCs and cDCs in WT and DNGR-1 KO (*Clec9a*^*Cre/Cre*^) at different time points after birth (*n* = 5–13 mice per time point). **(C)** Frequency of annexin V^+^ CDPs, pre-cDCs, and cDCs in BM and/or spleens of WT and DNGR-1 KO (*Clec9a*^*eGFP/eGFP*^) mice at 6 wk of age (*n* = 12 mice). **(D)** 6-wk-old mice were injected intraperitoneally with EdU 2 h prior to tissue harvest. Quantification of EdU^+^ cells in BM and spleen cDC progenitors and spleen cDCs of WT and DNGR-1 KO (*Clec9a*^*eGFP/eGFP*^) mice (*n* = 10–12 mice). Each dot in A and B represents averages, and data are pooled from three experiments; error bars represent SEM; (C and D) each dot represents one mouse, and data are pooled from two experiments. Bars represent averages and error bars represent SEM. Statistical significance in A and B was calculated using two-way ANOVA with Sidak’s multiple comparisons test, and in C and D was calculated using an unpaired *t* test with Welch’s correction. *P < 0.05 and **P < 0.01. EdU, 5-ethynyl-2′-deoxyuridine.

We next assessed whether DNGR-1 might have an additional role in cDC progenitors. As adult DNGR-1–deficient mice have a normal complement of cDCs (Fig. S1 I) (Salvermoser et al., 2018; Schraml et al., 2013), we focused on the postnatal period, when the cDC lineage is expanding. No differences in the total numbers of CDPs or pre-cDCs in BM were observed between DNGR-1 WT and KO mice in the first 5 wk of life (Fig. 2 A). However, in spleen, DNGR-1 KO pre-cDCs were more numerous than their WT counterparts in the first week after birth, although this difference was no longer evident after the second week (Fig. 2 B). Despite this, there were no significant differences in spleen cDC numbers between WT and DNGR-1 KO mice over the entire time course (Fig. 2 B). The frequency of apoptotic (annexin V^+^ DAPI^−^) CDPs, pre-cDCs, and cDCs in BM or spleen at day 42 after birth was also similar in WT and DNGR-1 KO mice (Fig. 2 C). In vivo 5-ethynyl-2′-deoxyuridine (EdU) incorporation analysis on postnatal day 42 failed to indicate marked differences between WT and DNGR-1 KO mice but did reveal a small increase in % EdU^+^ DNGR-1 KO pre-cDCs in BM (Fig. 2 D). Overall, these results indicate that DNGR-1 deficiency does not grossly affect cDCpoiesis but may have subtle effects in increasing pre-cDC proliferation and expansion, particularly in the early postnatal period.

### DNGR-1 expression impacts pre-cDCs colonization of peripheral tissues

**Figure 3.**
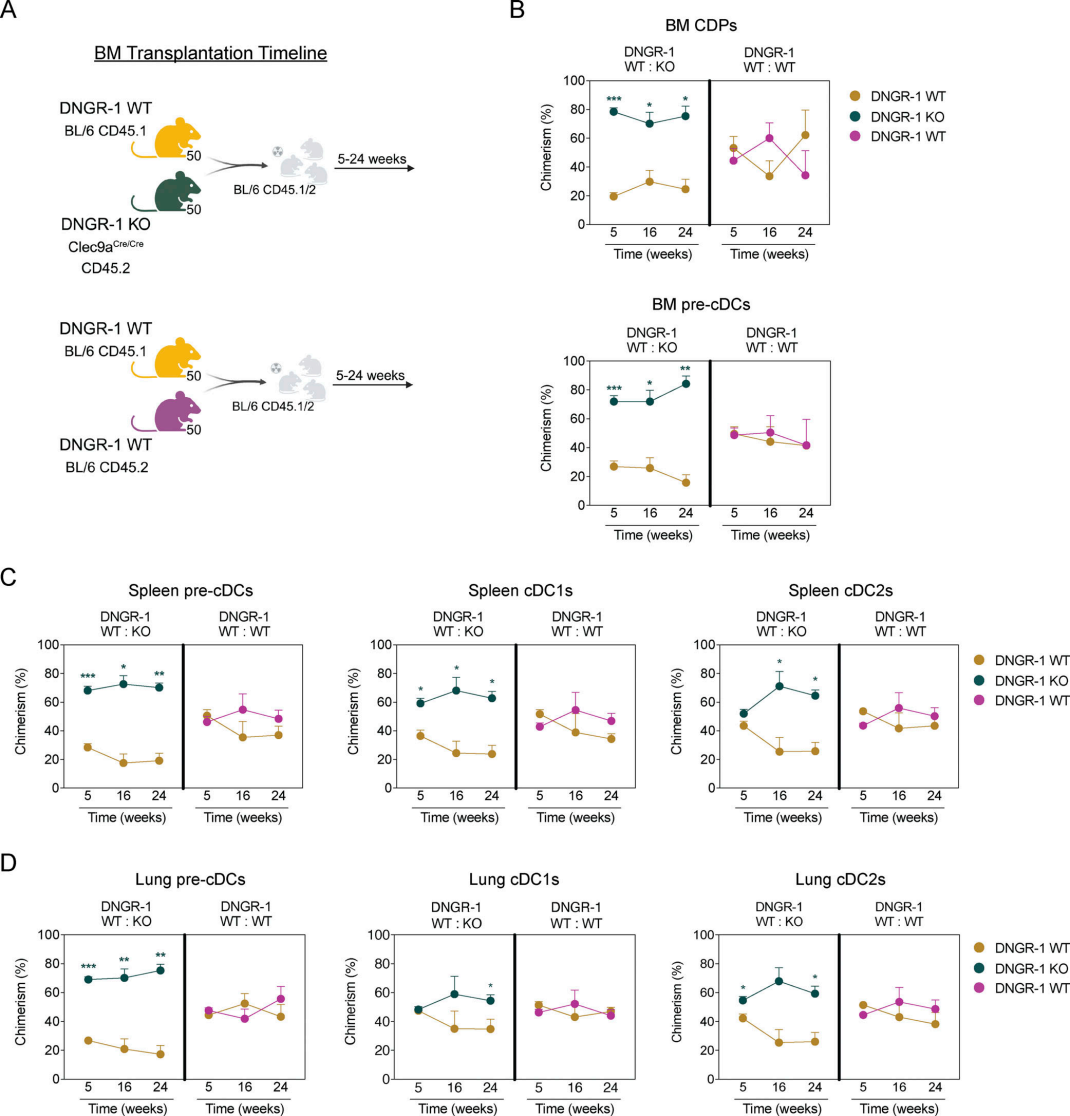
**DNGR-1 KO pre-cDCs are more efficient at peripheral tissue colonization. (A)** Lethally irradiated BL/6 CD45.1/2 recipients were reconstituted with a 1:1 ratio of either (a) WT (BL/6 CD45.1) and DNGR-1 KO (*Clec9a*^*Cre/Cre*^ CD45.2) or (b) WT (BL/6 CD45.1) and WT (BL/6 CD45.2) BM donor cells (2 × 10^6^ total). **(B–D)** Proportion of CDPs, pre-cDCs, and cDCs of WT:KO or WT:WT origin in (B) BM, (C) spleen, and (D) lung was measured over time (*n* = 4–5 mice per time point). Each dot in B–D represent averages, and error bars represent SEM. Data are representative from at least one experiment out of three. Statistical significance was calculated using two-way ANOVA with Sidak’s multiple comparisons test. *P < 0.05, **P < 0.01, and ***P < 0.001.

The finding that pre-cDCs numbers in spleen increase slightly more rapidly in DNGR-1 KO than in WT mice during early postnatal development could reflect their slightly elevated proliferation in BM (Fig. 2 D) and/or indicate a slight advantage of DNGR-1–deficient pre-cDCs in colonizing peripheral tissues. To probe this further, we generated radiation chimeric mice reconstituted with a 50:50 mix of BM cells from DNGR-1 WT (BL/6 CD45.1) and DNGR-1 KO (*Clec9a*^*Cre/Cre*^ CD45.2) mice (Fig. 3 A). In this competitive environment, even a small advantage in the proliferation, survival and/or migration of DNGR-1 KO cells would be magnified, as both sets of cells are vying for the same niche. Because CD45.1 and CD45.2 congenic mice possess additional genetic differences that could affect cell proliferation and migration (Chisolm et al., 2019), we generated a parallel set of control chimeras, mixing BM from WT BL/6 CD45.1 and WT BL/6 CD45.2 mice (Fig. 3 A). We then compared cDCpoiesis between WT:WT and WT:KO chimeras over time.

Analysis of mixed chimeras showed that the proportion of CDPs and pre-cDCs in the BM was significantly skewed toward cells of DNGR-1 KO origin as early as week 5 after transplantation (Fig. 3 B). Similarly, spleen and lung pre-cDCs populations were significantly biased toward cells derived from DNGR-1 KO donors (Fig. 3, C and D). These findings were consistent at 16 and 24 wk after transplantation, with a predominance of DNGR-1 KO pre-cDCs in the periphery, which correlated with a higher proportion of DNGR-1 KO differentiated cDC1 and cDC2s (Fig. 3, C and D). Because DNGR-1 is not expressed by cDC2s, the observed increase in that population indicates that changes in abundance over time are due to the activity of CDPs/pre-cDCs. Notably, no differences were observed in the ratio of WT:WT CDPs, pre-cDCs, or cDCs in the BM, spleen, or lung (Fig. 3, B–D), confirming that the differences in WT:KO chimeras were related to DNGR-1 status and not the cluster of differentiation 45 (CD45) congenic background. Altogether, these results suggest that DNGR-1 expression can adversely impact steady-state cDCpoiesis, slightly limiting CDP and pre-cDC numbers in BM and restraining peripheral tissue colonization by pre-cDC. Whether this involves ligand binding by DNGR-1 remains unresolved but seems unlikely. In the steady state, there is little exposure of F-actin, as most cell deaths occur by apoptosis, generating cell corpses that are rapidly scavenged or extruded from tissues before loss of membrane integrity (Bertheloot et al., 2021). Furthermore, secreted gelsolin, an abundant plasma protein, rapidly outcompetes DNGR-1 in binding to F-actin, creating a high threshold for DNGR-1 engagement that is only surpassed in pathological conditions (Giampazolias et al., 2021). Finally, pre-cDCs in tissues are likely to be outcompeted by more numerous cDC1s expressing higher levels of DNGR-1. Nevertheless, because chimeric mice were generated by whole body lethal irradiation, we cannot exclude that increased ligand exposure, from large-scale secondary necrosis of irradiated cells, contributed to the differences between DNGR-1–sufficient and –deficient cDC precursors in cDCpoiesis. Arguing against that notion, the differences in chimeric mice persisted for months after irradiation. Furthermore, deliberate treatment of WT versus DNGR-1 KO mice with ionizing radiation, myeloablative drugs, tissue damaging agents (e.g., bleomycin), or a variety of other insults designed to induce necrotic cell death failed to reveal differences in cDCpoiesis even when animals were crossed onto a secreted gelsolin–deficient background (unpublished data). Finally, preliminary data suggest that BM taken from mice bearing a mutant DNGR-1 that cannot bind ligand still displays a competitive advantage in contributing to cDCpoiesis in a mixed chimeric setting (not shown). Collectively, these data provide insights into the role of DNGR-1 in cDC progenitors, which helps our understanding of the kinetics of cDC replenishment in tissues. We have not studied the contribution of DNGR-1 to emergency cDCpoiesis, such as during acute infection, when increased cDC demand causes accelerated recruitment of pre-cDC from BM to tissues (Cabeza-Cabrerizo et al., 2021b; Pereira da Costa et al., 2023).

### DNGR-1 expression impacts pre-cDCs migratory pathways

**Figure 4.**
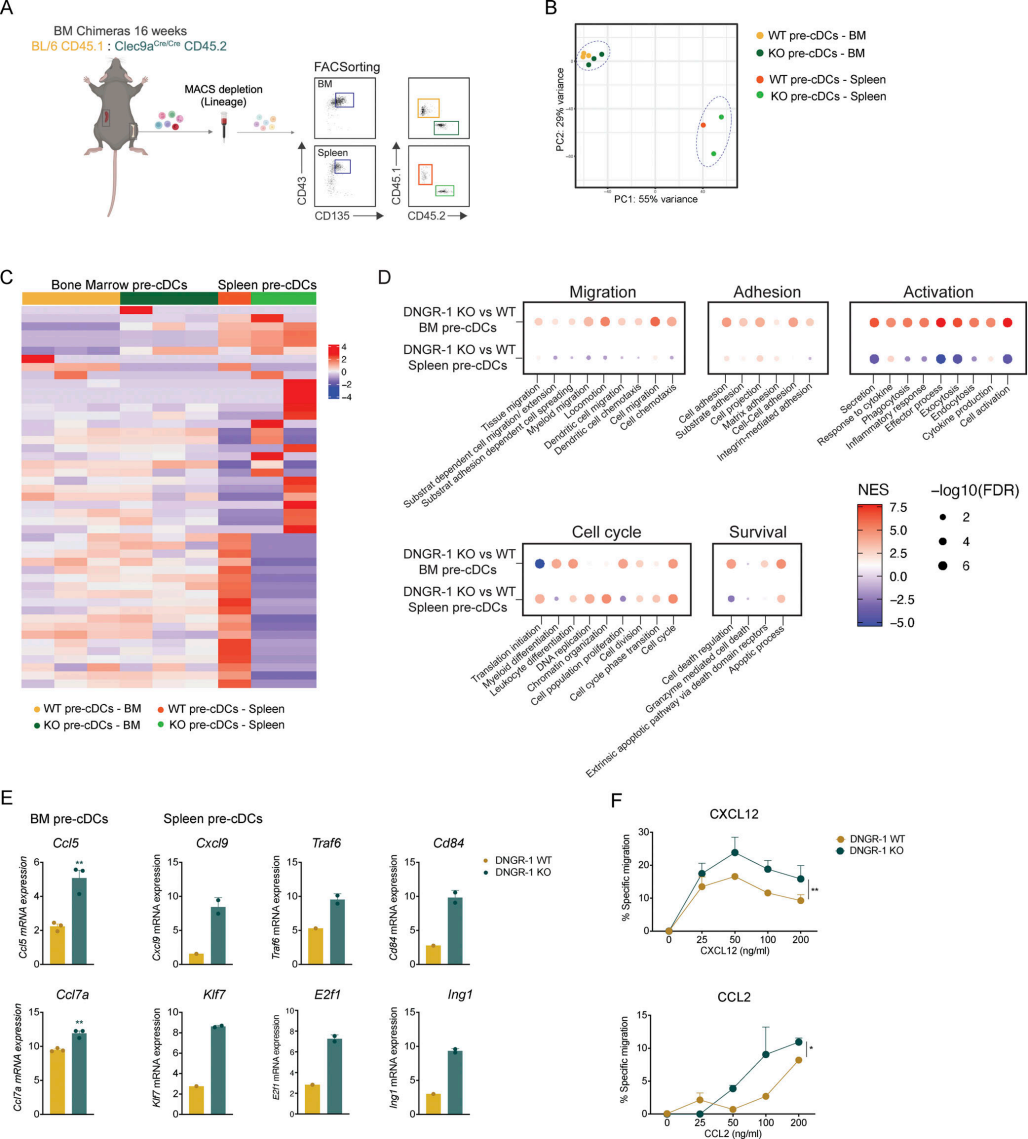
**DNGR-1 expression impacts gene expression signatures associated with pre-cDC migration. (A)** Bulk RNA-seq was performed on BM and splenic pre-cDCs isolated from mixed chimeric mice, with each sample pooled from two mice (*n* = 6 mice total). **(B)** Principal component analysis showing clustering of WT or DNGR-1 KO BM and splenic pre-cDCs. **(C)** Heatmap of the top 50 differentially expressed genes between KO and DNGR-1 WT pre-cDCs derived from BM or spleen. **(D)** Distribution of the top gene ontology biological processes in KO versus DNGR-1 WT pre-cDCs, in BM and spleen. **(E)** Differentially expressed genes in WT versus DNGR-1 KO pre-cDCs. **(F)** BM cells were seeded into the 96-well transwell inserts. After 2 h incubation, cells in the lower well were collected and re-stained. Frequency of BM pre-cDCs recruited toward CXCL12 or CCL2 as assessed by FACS (*n* = 6 biological replicates). Data are representative from at least one experiment out of three. Each dot in E and F represents a biological replicate; to achieve one biological replicate, cells from two to three mice were pooled. Bars represent averages, and error bars represent SEM. Statistical significance was calculated in E using unpaired *t* test with Welch’s correction, and in F using two-way ANOVA with Sidak’s multiple comparisons test. *P < 0.05 and **P < 0.01.

**Figure S2.**
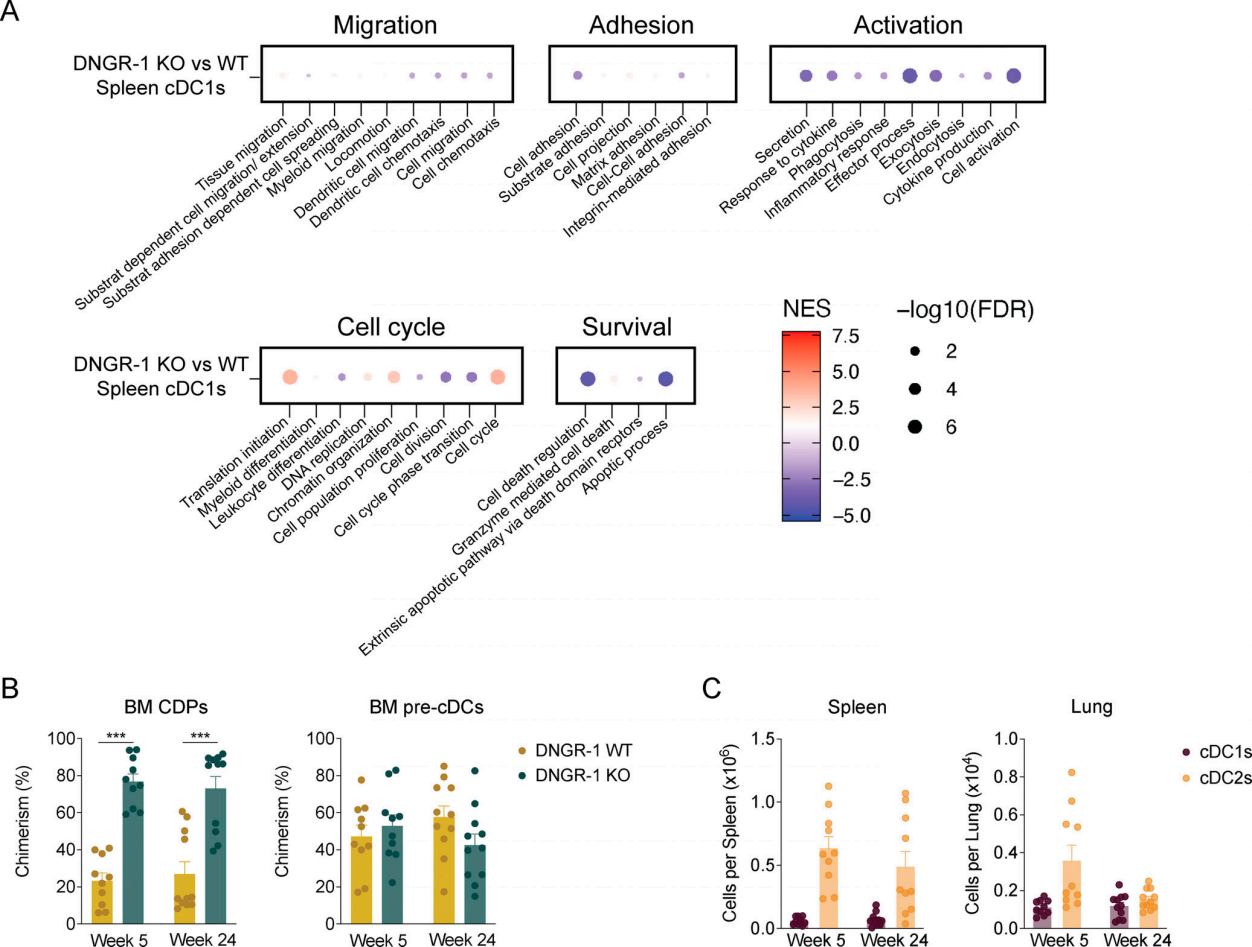
**DNGR-1 KO pre-cDCs are more efficient at peripheral tissues colonization than DNGR-1 WT pre-cDCs. (A)** Distribution of the top gene ontology biological processes in KO versus DNGR-1 WT cDC1s in spleen. Data are from the same mice shown in Fig. 4 D. **(B)** Frequency of BM CDPs and pre-cDCs at week 5 and 24 after transplantation (*n* = 10–11 mice). Data are from the same mice shown in Fig. 5 B, comparing WT and DNGR-1 KO frequencies over time. **(C)** Total number of spleen and lung cDC1s and cDC2s pre-cDCs in mixed BM chimeras. Data are from the same mice shown in Fig. 5 B, comparing WT and DNGR-1 KO frequencies over time. Each dot in A represents a biological replicate; to achieve one biological replicate, cells from two mice were pooled. Each dot in B and C represents one mouse, and data are from at least two experiments out of three. Bars represent averages, and error bars represent SEM. Statistical significance was calculated in B and C using two-way ANOVA with Sidak’s multiple comparisons test. ***P < 0.001.

To elucidate how DNGR-1 deficiency impacts cDCpoiesis, we focused on pre-cDC exit from the BM and migration to peripheral tissues. We performed bulk RNA-sequencing (RNA-seq) of FACS-purified WT and DNGR-1 KO pre-cDCs isolated from the BM and from the spleen, as well as of splenic cDC1s. We used mixed chimeric mice as cell sources to ensure focus on cell-intrinsic factors, pooled cells from two mice for each sample, and sequenced 1–3 samples per group (Fig. 4 A). We then focused analysis on the pre-cDC samples. Principal component and cluster analysis revealed that WT and DNGR-1 KO BM pre-cDCs clustered together but separately from DNGR-1 WT and KO spleen pre-cDCs, suggesting that most of the variance was due to tissue localization and/or differentiation state rather than DNGR-1 expression (Fig. 4 B). Indeed, comparison of DNGR-1 KO versus WT BM pre-cDCs showed few significant differences in individual gene expression (Fig. 4 C). However, gene ontology biological process categorization revealed that DNGR-1 KO pre-cDCs in BM and, to a lesser degree, in spleen displayed enhanced migration, adhesion, activation, and cell cycle gene expression signatures (Fig. 4 D). Tissue and cDC migration and cell and substrate adhesion pathways were enhanced in DNGR-1 KO BM pre-cDCs, which also appeared to be more differentiated as their expression of genes encoding cytokines and effector molecules were enhanced (Fig. 4 D). Conversely, DNGR-1 KO pre-cDCs in spleen displayed decreased activation and cell proliferation signatures (Fig. 4 D). In contrast to pre-cDCs, gene expression signatures relating to migration, adhesion, and cell cycle were not overly different between DNGR-1 KO and WT splenic cDC1s from BM chimeras (Fig. S2 A). Detailed analysis showed increased expression of *CCL5* in DNGR-1 KO pre-cDCs from BM (Fig. 4 E). On the other hand, DNGR-1 KO pre-cDCs from spleen displayed increased expression of C-X-C motif chemokine ligand (*CXCL*)*9*, tumor necrosis factor receptor associated factor 6, and CD84, among others (Fig. 4 E). Taken together, these results suggest that DNGR-1 absence in pre-cDCs does not affect chemokine receptor expression (not shown) but leads to an increase in gene expression signatures that relate to proliferation, migration, and motility, which could contribute to increased BM exit and colonization of peripheral tissues. Consistent with this notion, DNGR-1 KO BM pre-cDCs were more motile in response to CXCL12 and CCL2 than WT BM pre-cDCs (Fig. 4 F), two chemokines known to regulate pre-cDC retention and exit from BM, respectively (Pereira da Costa et al., 2023).

### DNGR-1 expression determines pre-cDCs localization in tissues

**Figure 5.**
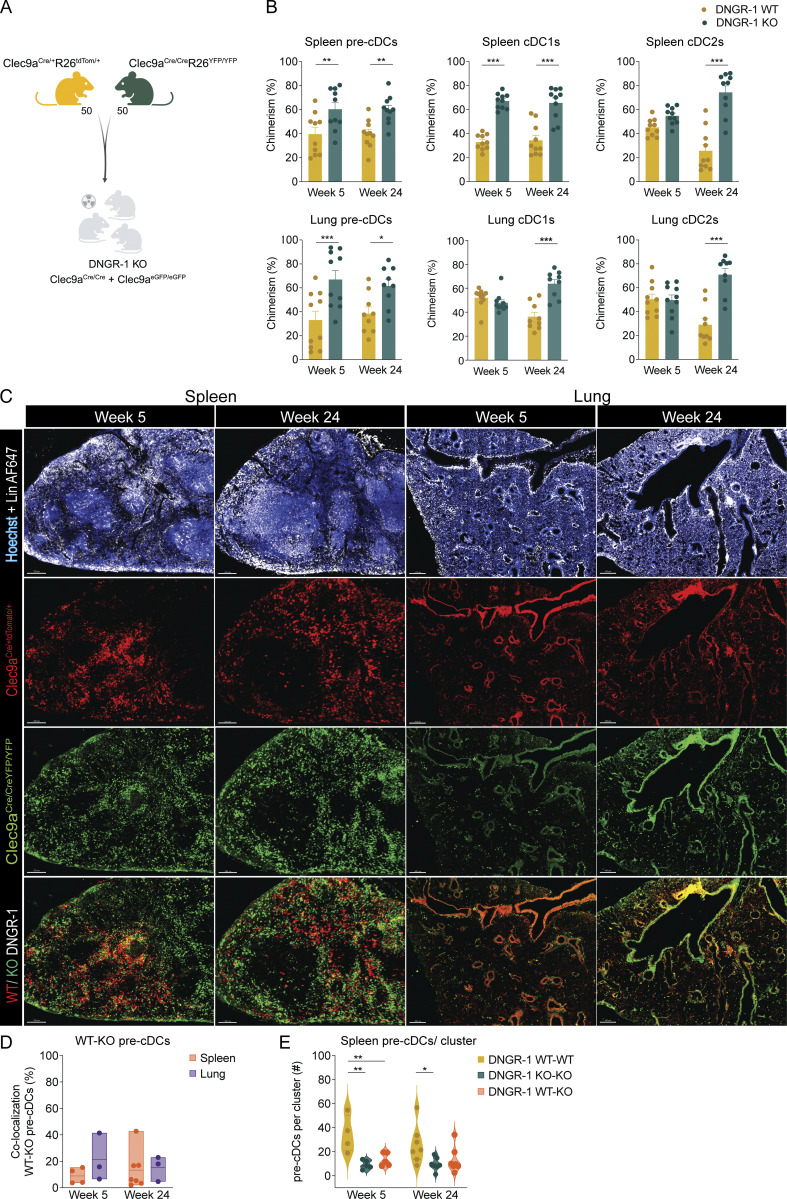
**DNGR-1 KO pre-cDCs are more efficient at peripheral tissues colonization. (A)** Lethally irradiated *Clec9a*^*Cre/Cre*^ and *Clec9a*^*eGFP/eGFP*^ recipients were reconstituted with a 1:1 ratio of *Clec9a*^*Cre/+*^*R26*^*tdTom/+*^ (WT) and *Clec9a*^*Cre/Cre*^*R26*^*YFP/YFP*^ (DNGR-1 KO) cells (2 × 10^6^ total donor cells). tdTom and YFP labeling in cDC subsets was normalized to maximum expression of respective donors. **(B)** Proportion of pre-cDCs and cDCs of WT or DNGR-1 KO origin in spleen (upper panels) and lung (lower panels) at 5 or 24 wk after reconstitution (*n* = 10–11 mice per time point). **(C)** Representative images of maximum projection of cryosections (35 µm) from WT:KO mixed chimeras. Spleen (left panels) and lung (right panels) sections (from 5 or 24 wk after reconstitution) were stained with Hoechst and AF647-labeled antibodies (anti–MHC class II, anti-CD64, and anti-B220) (upper). Middle panels show the localization of DNGR-1 WT (tdTom+ YFP−) and of DNGR-1 KO cDC subsets (tdTom− YFP+). **(D)** Co-localization of WT and DNGR-1 KO pre-cDCs in the spleen and lung at 5 or 24 wk after transplant. **(E)** Number of WT or DNGR-1 KO pre-cDCs per cluster in the spleen. See Materials and methods for details of analysis. Each dot in B represents one mouse, and data are pooled from at least two experiments out of three. Bars represent averages, and error bars represent SEM. Each dot in D and E corresponds to the analysis of one image from a whole spleen or lung taken from three to seven mice pooled from two experiments shown in B. Statistical significance was calculated in B, D, and E using two-way ANOVA with Sidak’s multiple comparisons test. *P < 0.05, **P < 0.01, and ***P < 0.001.

Given the subtle changes in migration suggested by the above analysis, we investigated the impact of DNGR-1 expression on pre-cDC/cDC tissue localization. We generated a separate set of radiation chimeric mice reconstituted with *Clec9a*^*Cre/+*^*R26*^*tdTom/+*^ (WT) mixed with *Clec9a*^*Cre/Cre*^*R26*^*YFP/YFP*^ (DNGR-1 KO) BM cells (Fig. 5 A). In this setting, WT pre-cDCs/cDCs are lineage traced with the reporter tdTom, while DNGR-1 KO cells are traced with the reporter YFP. Analysis showed that the proportion of cDCs and their precursors in BM and periphery was significantly biased toward YFP^+^ cells derived from DNGR-1 KO donors, recapitulating the analysis of the CD45 congenic mixed chimeras. (Fig. 5 B and Fig. S2 B). The kinetics of skewing toward the YFP^+^ population differed somewhat between spleen and lung and between pre-cDCs, cDC1s, and cDC2s (Fig. 5 B and Fig. S2 B), while the total numbers of splenic and lung cDCs remained unchanged over time (Fig. S2 C), suggesting that the initial colonization after transfer defined the cellular composition over time.

We prepared sections of spleen and lung from the chimeric mice to visualize the distribution of pre-cDCs in tissues, using a staining strategy previously developed for this purpose (Cabeza-Cabrerizo et al., 2021b; Pereira da Costa et al., 2023). Notably, this revealed a distinct distribution of WT tdTom^+^ and DNGR-1 KO YFP^+^ pre-cDCs, both in spleen and in lung (Fig. 5 C). Using histocytometry/CytoMAP tools (Stoltzfus et al., 2020), we observed that WT and DNGR-1 KO pre-cDCs are distributed differently in spleen (Fig. 5 D), with only 10–20% of the two genotypes displaying co-localization at the time points assessed (Fig. 5 D). Furthermore, density analysis revealed that DNGR-1 WT display a propensity to form higher density clusters than DNGR-1 KO pre-cDCs (Fig. 5 E), suggesting that while WT pre-cDCs remain grouped, DNGR-1 KO pre-cDCs have a greater propensity to spread throughout the tissue. Similar observations were made in the lung, where <30% of DNGR-1 WT and DNGR-1 KO pre-cDCs displayed co-localization at weeks 5 and 24 after transplantation (Fig. 5 D), although cluster analysis in that organ was not feasible due to the low numbers of cells present. Taken together, these data suggest that DNGR-1 expression in pre-cDCs influences their spatial distribution in peripheral tissues.

Despite the advancements made in this study, the precise mechanisms of action by which DNGR-1 affects cDC progenitor differentiation, migration, and localization remain elusive. One hypothesis is that DNGR-1 regulates pre-cDC behavior by subtly modulating the expression of receptors involved in migration and/or tissue retention, as suggested by gene expression analysis. Alternatively, DNGR-1 may influence the responsiveness of pre-cDCs to environmental cues that regulate migration and localization patterns. At present, we do not have a means of demonstrating whether DNGR-1–dependent restriction of cDCpoiesis has a functional impact. However, as cDC composition dictates both the quality and magnitude of the T cell response, elucidating the specific effects of DNGR-1 on the proliferative and migratory capabilities of pre-cDCs could offer an avenue for therapeutic interventions aimed at regulating the network of tissue cDCs.

## Materials and methods

### Resources

#### Lead contact

Further information and requests for resources should be directed to and will be fulfilled by the lead contact, Caetano Reis e Sousa (caetano@crick.ac.uk).

#### Material availability

All mouse lines generated in this study are available from the lead contact.

#### Data and code availability

Bulk RNA-seq dataset have been deposited at GEO at accession no. GSE278566. Any additional information required to reanalyze the data reported in this paper is available from the lead contact upon request.

### Experimental model

#### Mice

All mouse trains, C57BL/6J (BL/6; stock#000664; JAX), C57BL/6.SJL.CD45.1 (J) (BL/6 CD45.1; stock#002014; JAX), C57BL/6J xB6.SJL.CD45.1J F1 (BL/6 CD45.1/2), *Clec9a*^*Cre/Cre*^ (generated using BL/N-derived embryonic stem cells (ES) and backcrossed 10 times to C57BL/6J), *Clec9a*^*eGFP/eGFP*^ (generated using 129S6/C57BL/6 F1-derived ES cells and backcrossed 20 times to C57BL/6J), *Clec9a*^*tdTom*^ (generated using BL/N-derived ES cells and backcrossed 12 times to C57BL/6J), *Clec9a*^*Cre/Cre*^*R26*^*YFP/YFP*^ (ROSA26-EYFP backcrossed five times to *Clec9a*^*Cre/Cre*^), *Clec9a*^*Cre/+*^*R26*^*tdTom/+*^ (ROSA26-CAG-td Tomato crossed three times to *Clec9a*^*Cre/Cre*^; each colony crossed 10 times to C57BL/6J), and OT-I x *Rag1*^*−/−*^ were bred at the animal facility of the Francis Crick Institute. Mice were 6–12 wk of age at the start of the experiments unless otherwise stated. Mouse genotypes were determined using real-time PCR with specific probes designed for each gene (Transnetyx). Animal experiments were performed in accordance with national and institutional guidelines for animal care and were approved by the Francis Crick Institute Biological Resources Facility Strategic Oversight Committee (incorporating the Animal Welfare and Ethical Review Body) and by the Home Office, UK.

#### Cell lines

BRAF^V600E^ 5555 cells were grown in RPMI 1640 containing 10% FCS, 2 mM glutamine, 50 mM 2-mercaptoethanol, 100 U/ml penicillin, and 100 mg/ml streptomycin (R10). The cell line was independently screened negative for mycoplasma contamination by the Cell Services STP at the Francis Crick Institute.

### Methods

#### Preparation of single-cell suspensions

Tissues—femurs, tibias, spleens, LNs, and lungs—were recovered into FACS buffer (3% FCS and 5 mM EDTA in PBS). BM cells were extracted by flushing the femurs and tibias with 10 ml of FACS buffer. Spleens, LNs, and lungs were cut into small pieces and digested with collagenase VI (400 U/ml) and deoxyribonuclease I (0.4 mg/ml) in RPMI 1640 for 15–20 min (spleen and LNs) or 30–40 min (lung) at 37°C. Digested tissues were strained through a 70-μm cell strainer and washed with FACS buffer. For lung, leukocytes were enriched by Percoll gradient centrifugation. Blood leukocytes were enriched by Ficoll gradient centrifugation. BM cells, LNs, and lungs were resuspended in a final volume of 500 μl and spleen in a final volume of 3 ml.

#### BM transplantation assays

Lethally irradiated (6.6Gy twice with a 4-h interval between the two doses) BL/6 CD45.1/2 mice were grafted with (a) 2 × 10^6^ BM cells mix (1:1) of BL/6 5.1 and *Clec9a*^*Cre/Cre*^ 5.2 mice, (b) a mix (1:1) of BL/6 5.1 and BL/6 5.2 mice, or (c) a mix (1:1) of *Clec9a*^*Cre/+*^*R26*^*tdTom/+*^ and *Clec9a*^*Cre/Cre*^*R26*^*YFP/YFP*^. Donor reconstitution was assessed by peripheral blood analysis 4–5 wk after transplantation.

#### Migration assays

Flt3L (0.1 mg/mouse/day) was administered subcutaneously to BL/6 CD45.1 and *Clec9a*^*Cre*^ CD45.2 or *Clec9a*^*Cre/+*^*R26*^*tdTom/+*^ and *Clec9a*^*Cre/Cre*^*R26*^*YFP/YFP*^ mice for 4 consecutive days. At day 5, BM was isolated from femurs, tibias, and hipbones, and mature hematopoietic cells were excluded using magnetic depletion. BM pre-cDCs were further enriched by CD11c+ selection. The in vitro migration assay was performed using 96-well Transwell inlets with polycarbonate filters with 5-μm pore size. The transwells were preincubated with the chemokine in 235 μl complete medium in the lower well at 37°C, 5% CO_2_ in a humidified incubator 1 h before seeding the upper well. Increasing concentrations of CCL2 and CXCL12 were used. Complete medium without chemokine served as the negative control. 10^4^ total CD11c (“pre-cDCs”) enriched BM cells as described above were seeded in 80 μl complete medium to the inlet of the transwell and incubated at 37°C, 5% CO_2_ in a humidified incubator for 2 h. After 2 h, migrated cells were harvested from the lower well, labeled with pre-cDCs markers, and quantified by flow cytometry.

#### Flow cytometry and cell sorting

Cell suspensions were stained with antibodies listed in Table S1. Stained cells were analyzed on FACSymphony A5 or LSR Fortessa X20 or purified through a FACSAria III or FACS Aria Fusion (all from BD Biosciences). Data were analyzed on FlowJo software v10.7. Flow Cytometry Standard files were exported from FlowJo for analysis by uniform manifold approximation and projection performed in R (Becht et al., 2018). Quantitative flow cytometry analysis of surface DNGR-1 expression by spleen cDC1 (two experiments, *n* = 9 mice) was carried out as described (Fang et al., 2022) using calibration beads (BD Quantibrite Beads). By comparing the geometric mean fluorescence intensities from multiple experiments in which cDC1s and other cells were stained in parallel, surface receptor numbers were estimated and are depicted in Fig. S1 H.

#### Preparation of F-actin/myosin-coated beads

F-actin was prepared by mixing equal amounts (20 ml) of G-actin and biotinylated G-actin (both at 20 mM or 1 mg/ml), followed by addition of 5 ml of G-actin buffer and 5 ml of 103 F-actin buffer to start the polymerization reaction (1 h at room temperature [RT]). 12.5 ml of biotinylated F-actin (16 mM) was mixed with 27.5 ml of PBS and 10 ml of myosin II (20 mM or 10 mg/ml) for a final concentration of 4 mM each and incubated for 1 h at RT. Biotin–F-actin and myosin II was diluted 1:4 with PBS, and 100 ml was added to 20 ml of fluorescent or nonfluorescent streptavidin-coated beads, which had been washed twice with wash buffer (PBS + 1% BSA), for 30 min on ice. After washing, F-actin and myosin beads were resuspended with 100 ml of biotinylated OVA (0.4 mg/ml) and incubated for a further 30 min on ice. Washed beads were resuspended in wash buffer and sonicated (2 × 2 min) in a water bath sonicator before usage.

#### In vitro XP assay

Flt3L (0.1 mg/mouse/day) was administered subcutaneously to BL/6 or BL/6 and Clec9a^Cre^ mice for 4 consecutive days. At day 5, BM progenitors were isolated as before. Splenic cDCs were isolated by excluding other mature population through magnetic depletion and further positive selection of CD11c^+^ cells. BRAF^V600E^ 5555 cells were UV irradiated (240 mJ/cm^2^) and left overnight (ON) in serum-free RPMI 1640 medium. The following day, necrotic BRAF^V600E^ 5555 cells were pulsed with OVA (10 mg/ml) and poly(I:C) (1 µg/ml) for 1 h at 37°C and were washed three times in ice-cold PBS before resuspension in complete RPMI medium. BM progenitors or splenic cDCs were seeded at 2 × 10^4^ cells/well and OVA-pulsed dead cells were added at the indicated ratio and cultured in 96-well round-bottom plates at 37°C in RPMI 1640 medium containing 2 mM glutamine, 50 mM 2-mercaptoethanol, 100 U/ml penicillin, 100 mg/ml streptomycin, and 10% heat-inactivated FCS. To facilitate dead cell uptake, plates were centrifuged at 1,000 rpm for 3 min at the start of the incubation. Pre-activated OT-I T cells were added (5 × 10^4^/well) and co-cultured ON. Next morning, the plate was freeze-thawed once, and the total amount of IFN-γ was determined by ELISA.

#### Microscopy

Half of the spleen and lung (left lobe) were collected and placed directly into 4% PFA in PBS ON at 4°C. The following day, soft tissues were incubated in PBS with 30% sucrose at 4°C ON and embedded in Tissue-Tek OCT compound at −80°C after. The tissues were cut in a Leica 3050 cryostat to generate 30–40-μm frozen sections. For antibody staining, sections were hydrated in 0.1 M Tris (pH 7.4) and blocked for 1 h at 25°C in 1% BSA, 1% normal mouse serum, and 0.25% Triton TX-100. Sections were first stained ON at 4°C with rabbit anti-CD64, rat anti-CD45R/B220 biotin, rat anti–I-Ab biotin, and goat anti-eGFP diluted in blocking buffer. Sections were subsequently stained with donkey anti-rat AF647, donkey anti-rabbit AF647, and donkey anti-goat AF488. Sections were counterstained in Hoechst 33342 solution and mounted in ProLong Diamond Antifade. Imaging was performed in a Zeiss LSM 880 inverted confocal microscope with a 10x and a 25× oil immersion objective. Sequential excitation of fluorophores at 405, 488, 561, and 633 nm was provided by a combination of argon and helium lasers. Tile scans were acquired covering the entire surface area of the section at a step size of 2 μm and a pinhole of 1 Airy unit. Images were acquired with 512 × 512-pixel resolution with a line averaging of four. Tile stitching was performed using ZEN software (Zeiss).

#### Pre-cDCs localization analysis

Mapping of cDC progenitors in the spleen and lung was performed using a previously described approach (Cabeza-Cabrerizo et al., 2021b; Pereira da Costa et al., 2023). To exclude lineage^+^ cells, histocytometry was used (Gerner et al., 2012). Briefly, after generating surfaces using Imaris, values such as position (X, Y, and Z) or mean fluorescence intensity of different channels are exported into a csv file. The file was then imported into FlowJo, and individual precursor cells quantified using a gating-based strategy. The new analysis file was then run using CytoMAP for clustering analysis (Stoltzfus et al., 2020). CytoMAP generates neighborhoods of cells of a radius of 50 μm, based on the cellular composition of the neighborhood in terms of different cell densities. The number of regions were defined as 3: tdTom+ cells, YFP+ cells, and tdTom+ YFP+ co-localized cells.

#### RNA extraction

Sorted samples were isolated directly into lysis buffer of RNeasy Micro Kit and kept at −80°C until RNA extraction. mRNA, from CDPs, pre-cDCs and cDCs, was extracted using RNeasy Micro kit on the QIACube (Qiagen).

#### Bulk RNA-seq

pre-cDCs and cDCs were isolated from BM and spleen or spleen alone (respectively), using a BD FACSAria Aria II or Fusion sorter. Cells (1 × 10^4^–2 × 10^4^) were sorted directly into lysis buffer to avoid loss of material. RNA was extracted as described above. Sequencing was performed by the Beijing Genomics Institute using DNBSEQ next generation sequencing technology and analysis done in-house. As Ly5.1– and Ly5.2 derived–animals possess intrinsic genetic differences that may affect cell proliferation and migration, a set of 205 genes on chromosome 1 between 106465908 and 143697024 previously described to impact these pathways (Chisolm et al., 2019), were used to score the cells and regress out effects dominated by the background effect. Differentially expressed genes were defined as those showing statistically significant differences between the DNGR-1 KO and DNGR-1 WT groups. Differential genes were ranked by their log_2_FC value and used for gene set enrichment analysis using hallmark, pathways and processes gene sets from MsigDB (v6) (Subramanian et al., 2005).

#### Quantification and statistical analysis

Statistical significance was determined using Student’s *t* test, one-way ANOVA, or two-way ANOVA with post hoc testing as indicated in the figure legends. Outliers were identified using ROUT. Graphs containing errors bars show means ± SD or SEM. Statistically significant values are indicated in the figures. These tests were performed with Prism Software (GraphPad).

### Online supplemental material

Fig. S1 shows the flow cytometry analysis of mouse cDCs and their progenitors and the expression of the DNGR-1 receptor on these subsets. Fig. S2 shows the DNGR-1 KO pre-cDCs efficiency at colonizing peripheral tissues in a BM chimeric setting. Table S1 lists the reagents and resources used for the experiments reported in this manuscript.

## Supplementary Material

Table S1 shows the list of reagents or resources used for the experiments reported in this manuscript.

## Data Availability

RNA-seq data can be found at NCBI Gene Expression Omnibus repository (accession number GSE278566). Additional data are available in the article itself and its supplementary materials and are also available upon reasonable request from the corresponding author.
