# Supplementary material for: DNGR-1 regulates proliferation and migration of bone marrow dendritic cell progenitors
Source: J Exp Med. 2025 May 13;222(8):e20241813. doi: 10.1084/jem.20241813 (PMC12071193; doi:10.1084/jem.20241813)
Supplement: Table S1 — shows the list of reagents or resources used for the experiments reported in this manuscript. [file jem_20241813_tables1.docx]

**Table S1. List of** **reagents or resources used for the experiments reported in this manuscript**

| **REAGENT or RESOURCE** | **SOURCE** | **IDENTIFIER** |
| --- | --- | --- |
| **Antibodies** | | |
| AF647 anti-DNGR-1 (clone 1F6) | Francis Crick Institute | N/A |
| AF647 anti-mouse CD11b (clone M1/70) | Biolegend | Cat# 101218; RRID:AB_389327 |
| AF647 anti-mouse CD45R/B220 (clone RA3-6B2) | Biolegend | Cat# 103226; RRID:AB_389330 |
| AF647 anti-mouse I-A/I-E (clone M5/114.15.2) | Biolegend | Cat# 107618; RRID:AB_493525 |
| AF700 anti-mouse CD34 (clone RAM34) | Fisher Scientific | Cat# **56-0341-82** |
| AF700 anti-mouse MHC Class II (I-A/I-E; clone M5/114.15.2) | Thermos Fisher | Cat# 56-5321-82; RRID:AB_494009 |
| APC anti-mouse CD45.1 (clone A20) | Fisher Scientific | Cat# 17-0453-82; RID:AB_493998 |
| APC anti-mouse CD48 (clone HM48-1) | Biolegend | Cat# 103412; RRID:AB_571997 |
| APC eFluor780 anti-mouse CD11b (clone M1/70) | Fisher Scientific | Cat# 47-0112-82; RRID:AB_1603193 |
| APC-eFluor 780 anti-mouse CD11c (clone N418) | Fisher Scientific | Cat# 47-0114-82; RRID:AB_1548652 |
| APC-eFluor™ 780 anti-mouse CD64 (clone X54-5/7.1) | Fisher Scientific | Cat# 47-0641-82; RRID:AB_2735012 |
| APC/Cyanine7 anti-mouse CD117 (clone 2B8) | Biolegend | Cat# 105826; RRID:AB_1626278 |
| APC/Cyanine7 anti-mouse CD16/32 (clone | Biolegend | Cat# 101328; RRID:AB_2104158 |
| APC/Fire 750 anti-mouse CD48 (clone HM48-1) | Biolegend | Cat# 103446; RRID:AB_2650847 |
| APC/Fire™ 810 anti-mouse CD19 clone 6D5) | Biolegend | Cat# 115577; RRID:AB_2892274 |
| BD Horizon™ BUV563 Streptavidin | BD Biosciences | Cat# 612935; RRID:AB_2870219 |
| BD Horizon™ BUV737 Streptavidin | BD Biosciences | Cat# 612775; RRID:AB_2870104 |
| Biotin anti-mouse CD11c (clone N418) | Biolegend | Cat# 117304; RRID:AB_313773 |
| Biotin anti-mouse CD170 (clone S17007L) | Biolegend | Cat# 155512; RRID:AB_2814066 |
| Biotin anti-mouse CD19 (clone 6D5) | Biolegend | Cat# 115504; RRID:AB_313639 |
| Biotin anti-mouse CD3ε (clone 145-2C11) | Biolegend | Cat# 100304; RRID:AB_312669 |
| Biotin anti-mouse CD4 (clone GJ1.5) | Biolegend | Cat# 100404; RRID:AB_312689 |
| Biotin anti-mouse CD8a (clone 53-6.7) | Biolegend | Cat# 100704; RRID:AB_312743 |
| Biotin anti-mouse Gr1 (clone RB6-8C5) | Biolegend | Cat# 108404; RRID:AB_313369 |
| Biotin anti-mouse MHC Class II (clone M5/114.15.2) | Biolegend | Cat# 107604; RRID:AB_313319 |
| Biotin anti-mouse Ly-6C (clone HK1.4) | Biolegend | Cat# 128004; RRID:AB_1236553 |
| Biotin anti-mouse Ly-6D REAfinity™ (clone REA906) | Miltenyi | Cat# 130-115-310; RRID:AB_2726990 |
| Biotin anti-mouse Ly-6G (clone 1A8) | Biolegend | Cat# 127604; RRID:AB_1186108 |
| Biotin anti-mouse NK1.1 (clone PK136) | Biolegend | Cat# 108704; RRID:AB_313391 |
| Biotin anti-mouse Ter119 (clone TER-119) | Biolegend | Cat# 116204; RRID:AB_313705 |
| Biotin anti-mouse B220 (clone RA3-6B2) | Biolegend | Cat# 103204; RRID:AB_312989 |
| Biotin anti-mouse/human CD11b (clone M1/70) | Biolegend | Cat# 101204; RRID:AB_312787 |
| Brilliant Violet 421 anti-mouse XCR1 (clone TER-119) | Biolegend | Cat# 148216; RRID:AB_2565230 |
| Brilliant Violet 421 anti-mouse CD170 (clone E50-2440) | BD Biosciences | Cat# 562681; RRID:AB_2722581 |
| Brilliant Violet 421™ anti-mouse CD11c (clone N418) | Biolegend | Cat# 117343; RRID:AB_2563099 |
| Brilliant Violet 421™ anti-mouse CD19 (clone (6D5) | Biolegend | Cat# 115549; RRID:AB_2563066 |
| Brilliant Violet 421™ anti-mouse MHC Class II (clone M5/114.15.2) | Biolegend | Cat# 107632; RRID:AB_2650896 |
| Brilliant Violet 510™ anti-mouse CD172a (SIRPα) | Biolegend | Cat# 144032; RRID:AB_2810411 |
| Brilliant Violet 605 anti-mouse Sca1 (clone D7) | Biolegend | Cat# 108133, RRID:AB_2562275 |
| Brilliant Violet 605™ anti-mouse CD115 (clone AFS98) | Biolegend | Cat# 135517; RRID:AB_2562760 |
| Brilliant Violet 605™ anti-mouse Ly-6C (clone HK1.4) | Biolegend | Cat# 128036; RRID:AB_2562353 |
| Brilliant Violet 605™ anti-mouse CD11b (clone M1/70) | Biolegend | Cat# 101257; RRID:AB_2565431 |
| Brilliant Violet 650 anti-mouse CD45R/B220 (clone RA3-6B2) | Biolegend | Cat# 103241; RRID:AB_11204069 |
| Brilliant Violet 650™ anti-mouse CD11c (clone N418) | Biolegend | Cat# 117339; RRID:AB_2562414 |
| Brilliant Violet 711 anti-mouse CD150 (cloneTC15-12F12.2) | Biolegend | Cat# 115941; RRID:AB_2629660 |
| Brilliant Violet 711 anti-mouse CD170 (Siglec-F; clone E50-2440) | BD Biosciences | Cat# 740764; RRID:AB_2740427 |
| Brilliant Violet 785 anti-mouse CD3ε (clone 145-2C11) | Biolegend | Cat# 100355; RRID:AB_2565969 |
| Brilliant Violet 785™ anti-mouse CD127 (IL-7Rα; clone A7R34) | Biolegend | Cat# 135037; RRID:AB_2565269 |
| Brilliant Violet 785™ anti-mouse XCR1 (clone ZET) | Biolegend | Cat# 148225; RRID:AB_2783119 |
| BUV395 anti-mouse CD11b (clone M1/70) | BD Biosciences | Cat# 563553; RRID:AB_2738276 |
| BUV563 anti-mouse CD115 (CSF-1R) (clone T38-320) | BD Biosciences | Cat# 748478; RRID:AB_2872893 |
| BUV563 anti-mouse CD16/CD32 (clone 2.4G2) | BD Biosciences | Cat# 741229; RRID:AB_2870783 |
| BUV661 anti-mouse CD45.2 (clone 30F11) | BD Biosciences | Cat# 741516; RRID:AB_2870965 |
| BUV661 anti-mouse CD45R/B220 (clone RA3-6B2) | BD Biosciences | Cat# 565077; RRID:AB_2739056 |
| BUV805 anti-mouse CD11c (clone N418) | BD Biosciences | Cat# 749038; RRID:AB_2873432 |
| BV510 Rat Anti-mouse CD115 (clone T38-320) | BDBiosciences | Cat# 743639; RRID: AB_2741649 |
| Donkey anti-Goat IgG (H+L) PLUS AF488 | Thermo Fisher | Cat# A32814 |
| Donkey anti-Rabbit IgG (H+L) PLUS AF647 | Thermo Fisher | Cat# A32795 |
| Donkey anti-Rat IgG (H+L) PLUS AF647 | Thermo Fisher | Cat# A48272 |
| eFluor 450 anti-mouse CD11b (clone M1/70) | Fisher Scientific | Cat# 48-0112-82; RRID:AB_1582236 |
| eFluor 450 anti-mouse CD34 (clone RAM34) | Fisher Scientific | Cat# 48-0341-82; RRID:AB_2043837 |
| eFluor 450 anti-mouse Ly-6D (clone 49-H4) | Fisher Scientific | Cat# 48-5974-80; RRID:AB_2574089 |
| eFluor 450 anti-mouse/ human CD45R/B220 (clone RA3-6B2) | Fisher Scientific | Cat# 48-0452-82; RRID:AB_1548761 |
| eFluor™ 450 anti-mouse CD3e (clone 145-2C11) | Fisher Scientific | Cat# 48-0031-82; RRID:AB_10735092 |
| FITC anti-mouse CD170 (Siglec-F; clone S17007L) | Biolegend | Cat# 155504; RRID:AB_2750233 |
| FITC anti-mouse CD19 (clone 6D5) | Biolegend | Cat# 115506; RRID:AB_313641 |
| FITC anti-mouse CD3ε (clone 145-2C11) | BD Pharmigen | Cat# 553062; RRID:AB_394595 |
| FITC anti-mouse CD4 (clone GK1.5) | Biolegend | Cat# 100406; RRID:AB_312691 |
| FITC anti-mouse CD43 Activation-Associated Glycoform (clone 1B11) | Biolegend | Cat# 121206; RRID:AB_493386 |
| FITC anti-mouse CD45R/B220 (clone RA3-6B2) | BD Pharmigen | Cat# 553088 |
| FITC anti-mouse CD8a (clone 53-6.7) | Biolegend | Cat# 100706; RRID:AB_312745 |
| FITC anti-mouse Ly-6D (clone 49-H4) | Biolegend | Cat# 138606; RRID:AB_11203888 |
| FITC anti-mouse Ly-6G (clone 1A8) | Biolegend | Cat# 127606; RRID:AB_1236494 |
| FITC anti-mouse Ly-6G/Ly-6C (Gr1; clone RB6-8C5) | Biolegend | Cat# 108406, RRID:AB_313371 |
| FITC anti-mouse NK-1.1 (clone PK136) | Biolegend | Cat# 108706; RRID:AB_313393 |
| FITC anti-mouse Sca1 (clone D7) | Biolegend | Cat# 108106; RRID:AB_313343 |
| FITC anti-mouse TER-119 (clone TER-119) | Biolegend | Cat# 116206; RRID:AB_313707 |
| Goat anti-eGFP | Abcam | Cat# ab6673 |
| Goat anti-mouse IgG Biotin ELISA detection | SouthernBiotech | Cat# 1030-08 RRID: AB_2794296 |
| Pacific Blue anti mouse NK1.1 (clone PK136) | Biolegend | Cat# 108722; RRID:AB_2132712 |
| Pacific Blue anti-mouse Ly-6G (clone 1A8) | Biolegend | Cat# 127612; RRID:AB_2251161 |
| Pacific Blue™ anti-mouse CD4 (clone GK1.5) | Biolegend | Cat# 100428; RRID:AB_493647 |
| Pacific Blue™ anti-mouse CD8a (clone 53-6.7) | Biolegend | Cat# 100725; RRID:AB_493425 |
| Pacific Blue™ anti-mouse Ly-6G/Ly-6C (Gr-1; clone RB6-8C5) | Biolegend | Cat# 108430; RRID:AB_893556 |
| Pacific Blue™ anti-mouse TER-119 (clone TER-119) | Biolegend | Cat# 116232; RRID:AB_2251160 |
| PE anti-DNGR-1 (clone 1F6) | Francis Crick Institute | N/A |
| PE anti-mouse CD135 (clone A2F10) | Biolegend | Cat# 135306; RRID:AB_1877217 |
| PE anti-mouse CD16/32 (clone 93) | Biolegend | Cat# 101308; RRID:AB_312807 |
| PE anti-mouse CD45.2 (clone 104) | Biolegend | Cat# 109808; RRID:AB_313445 |
| PE anti-mouse Ly-6C (clone HK1.4) | Biolegend | Cat# 128008; RRID:AB_1186132 |
| PE/Cyanine7 anti-mouse CD117 (clone | Biolegend | Cat# 105814; RRID:AB_313223 |
| PE/Cyanine7 anti-mouse CD127 (clone A7R34) | Biolegend | Cat# 135014; RRID:AB_1937265 |
| PE/Cyanine7 anti-mouse CD19 (clone 6D5) | Biolegend | Cat# 115520; RRID:AB_313655 |
| PE/Cyanine7 anti-mouse CD43 Activation-Associated Glycoform (clone 1B11) | Biolegend | Cat# 121218; RRID:AB_528813 |
| PE/Cyanine7 anti-mouse CD45.2 (clone 104) | Biolegend | Cat# 109830; RRID:AB_1186098 |
| PE/Cyanine7 anti-mouse CD64 (clone X54-5/7.1) | Biolegend | Cat# 139314; RRID:AB_2563904 |
| PerCP-eFluor 710 anti-mouse CD135 (clone A2F10) | Fisher Scientific | Cat# 46-1351-82; RRID:AB_10733393 |
| PerCP/Cy5.5 anti-mouse CD11c (clone N418) | Biolegend | Cat# 117328; RRID:AB_2129641 |
| PerCP/Cyanine5.5 anti-mouse CD16/32 (clone 93) | Biolegend | Cat# 101324; RRID:AB_1877267 |
| PerCP/Cyanine5.5 anti-mouse Gr1 (clone RB6-8C5) | Biolegend | Cat# 108428; RRID:AB_893558 |
| PerCP/Cyanine5.5 anti-mouse Ly-6G (clone 1A8) | Biolegend | Cat# 127616; RRID:AB_1877271 |
| PerCP/Cyanine5.5 anti-mouse/human CD45R/B220 (clone RA3-6B2) | Biolegend | Cat# 103236; RRID:AB_893354 |
| Purified CD16/32 (FcBlock; (clone 24G2) | BD | Cat# 553141; RRID:AB_394656 |
| Rabbit anti-mouse CD64 | Stratech Scientific | Cat# 50086-R027 |
| Rat anti-mouse IFN-g ELISA capture (R4-6A2) | BD Biosciences | Cat# 551216; RRID:AB_394094 |
| Rat anti-mouse IFN-g ELISA detection (XMG1.2) | BD Biosciences | Cat# 554410; RRID:AB_395374 |
| Streptavidin eFluor™ 450 conjugate | Thermo Fisher | Cat# 48-4317-82; RRID:AB_10359737 |
| Streptavidin Alexa Fluor™ 488 conjugate | Biolegend | Cat# S32354 |
| Super Bright 702 anti-mouse CD117 (clone 2B8) | Fisher Scientifc | Cat# 67-1171-82; RRID:AB_2717159 |
| Super Bright™ 600 anti-mouse CXCR4 (clone 2B11) | Fisher Scientific | Cat# 63-9991-82; RRID:AB_2688143 |
|  |  |  |
| **Chemicals, peptides, and recombinant proteins** | | |
| Actin biotin-conjugated | Cytoskeleton Inc | AB07 |
| Actin from skeletal muscle | Cytoskeleton Inc. | AKL99 |
| Actin Polymerization buffer (10x) | Cytoskeleton Inc | BSA02-001 |
| Alexa Fluor 647 Annexin V | Biolegend | Cat# 640911 |
| Annexin V Binding Buffer | Biolegend | Cat# 422201 |
| Collagenase IV | Worthington | Cat# LS004188 |
| DNAse I | Roche | Cat# 11284932001 |
| ExtrAvidin-Alkaline Phosphatase | Sigma | E2636 |
| Ficoll-Paque PREMIUM 1.084 | Fisher Scientific | Cat# 17-5446-02 |
| Fixation Medium A | Nordic MUbio | Cat# GAS-002A-1 |
| InVivoMAb recombinant Flt-3L-Ig (hum/hum) | BioXcell | Cat#BE0098-1mg |
| Myosin II from rabbit skeletal muscle | Cytoskeleton Inc. | MY02 |
| OVA peptide (SIINFEKL) | Francis Crick Institute | N/A |
| Paraformaldehyde 16% solution, EM Grade | Electron microscopy sciences | Cat# 15710 |
| Percoll | GE Healthcare | Cat# 17-0891-01 |
| Recombinant Murine JE/MCP-1 (CCL2) | Peprotech | Cat# 250-10 |
| Recombinant Murine SDF-1a (CXCL12) | Peprotech | CAT# 250-20A |
| Tissue Tek OCT Compound | Sakura Finetek | Cat# 4583 |
| Triton X-100 for molecular biology | Sigma | Cat# T8787-250mL |
|  |  |  |
| **Critical commercial assays** | | |
| Anti-biotin microbeads | Miltenyi Biotec | Cat# 130-090-485 |
| Anti-FITC microbeads | Miltenyi Biotec | Cat# 130-048-701 |
| CD11c microbeads | Miltenyi Biotec | Cat# 130-125-835 |
| Click-iT EdU Cell Proliferation | Invitrogen | Cat# C10340 |
| BD Quantibrite™ Beads | BD | Cat# 34049 |
| FoxP3/Transcription Factor staining buffer | Thermo Fisher | Cat# 00-5523-00 |
| HTS Transwell-96 Permeable Support (5µm) | Corning | Cat# CLS3387-8EA |
| LIVE/DEAD Fixable Aqua Dead Cell Stain | Thermo Fisher | Cat# L34966 |
| LIVE/DEAD Fixable Blue Dead Cell Stain | Thermo Fisher | Cat# L34962 |
| RNeasy Micro kit | Qiagen | Cat# 74004 |
|  |  |  |
| **Deposited data** | | |
| Bulk RNA sequencing of BL/6 WT and *Clec9a^Cre^* BM and spleen pre-cDCs, and spleen cDC1s | This paper (NCBI Gene  Expression Omnibus) | GSE278566 |
|  |  |  |
| **Experimental models: Cell lines** | | |
| BRAFV600E 5555 | Gift from Richard Marais (University of Manchester, Manchester, UK) | N/A |
|  |  |  |
| **Experimental models: Organisms/strains** | | |
| (C57BL/6J xB6.SJL.CD45.1J) F1 | Francis Crick Institute | Generated in-house |
| C57BL/6.SJL.CD45.1 (J) | Francis Crick Institute | RRID:IMSR_JAX:002014 |
| C57BL/6J | Francis Crick Institute | RRID:IMSR_JAX:000664 |
| *Clec9a^Cre^* | Francis Crick Institute | N/A |
| *Clec9a^Cre^Rosa^LSLEYFP^* | Francis Crick Institute | N/A |
| *Clec9a^Cre^Rosa^LSLtdTomato^* | Francis Crick Institute | N/A |
| *Clec9a^eGFP^* | Francis Crick Institute | N/A |
| *Clec9a^tdTomato^* | Francis Crick Institute | N/A |
| OT-I *Rag1^-/-^* | Francis Crick Institute | N/A |
|  |  |  |
| **Software and algorithms** | | |
| Adobe Illustrator | Adobe System | https://www.adobe.com/ |
| Biorender | Biorender | https://biorender.com/ |
| Dimensionality reduction for visualizing single-cell data using UMAP (R package) | Bech, 2018 | https://codeload.github.com/lmcinnes/umap/tar.gz/0.2.4 |
| FIJI (v2.1.0) | ImageJ | https://imagej.nih.gov/ij/ |
| FlowJo (v10.8.1) | FlowJo LLC | https://www.flowjo.com |
| Imaris (v.9.1.2) | Imaris Software | https://imaris.oxinst.com/ |
| Prism (GraPad 9.2.0) | GraphPad Software | https://www.graphpad.com |
| R Studio (v3.5) | R Studio | https://www.rstudio.com |
| ZEN black | Zeiss | https://www.zeiss.com/microscopy/int/products/microscope-software/zen.html |
